# Supplementary material for: Assessing COVID-19-Related Excess Mortality Using Multiple Approaches—Italy, 2020–2021
Source: Int J Environ Res Public Health. 2022 Dec 17;19(24):16998. doi: 10.3390/ijerph192416998 (PMC9779266; doi:10.3390/ijerph192416998)
Supplement: Supplementary file 1 [file ijerph-19-16998-s001.zip › Supplementary materials - table S1.pdf]

Supplementary Table S1.a Excess mortality estimates in 2020 in the different models by region.

	ISTAT-ISS REPORT 2020	Model 1 - 2020	Model 2 -2020	Model 3 - 2020
**Abruzzo**	943	1228 (623; 1789)	941 (906, 974)	1802 (1285, 2292)
**Basilicata**	346	275 (-69; 592)	346 (339, 356)	553 (301, 778)
**Calabria**	814	783 (-30; 1538)	815 (789, 848)	1339 (632, 1972)
**Campania**	3907	4563 (2668; 6343)	3925 (3780, 4064)	4980 (3174, 6662)
**Emilia-Romagna**	8762	8699 (7120; 10177)	8761 (8515, 9012)	10752 (9127, 12276)
**Friuli-Venezia Giulia**	1843	1978 (1448; 2475)	1848 (1769, 1935)	2435 (1919, 2897)
**Lazio**	3233	3631 (1565; 5564)	3241 (3141, 3335)	5059 (3211, 6812)
**Liguria**	3719	4046 (3248; 4787)	3724 (3569, 3872)	4799 (4082, 5461)
**Lombardia**	36500	34887 (31290; 38280)	36354 (34427, 38398)	39484 (36112, 42538)
**Marche**	2260	2482 (1675; 3223)	2258 (2159, 2358)	3018 (2411, 3558)
**Molise**	230	300 (62; 517)	232 (222, 239)	418 (262, 561)
**Piemonte**	12306	12232 (10179; 14169)	12378 (11661, 13145)	14035 (12253, 15668)
**P.A. Bolzano**	1011	978 (710; 1221)	1011 (960, 1066)	1010 (828, 1176)
**P.A. Trento**	1526	1474 (1192; 1734)	1526 (1440, 1608)	1690 (1491, 1872)
**Puglia**	4815	4713 (3107; 6214)	4811 (4605, 5028)	4854 (3507, 6092)
**Sardegna**	2158	1785 (1049; 2470)	2156 (2109, 2214)	1864 (1242, 2438)
**Sicilia**	3114	2897 (1078; 4614)	3113 (2983, 3265)	4163 (2403, 5822)
**Toscana**	3824	3970 (2427; 5403)	3824 (3700, 3954)	4744 (3309, 6099)
**Umbria**	586	692 (200; 1145)	590 (567, 616)	962 (583, 1303)
**Valle d'Aosta**	368	411 (275; 533)	368 (341, 399)	372 (293, 442)
**Veneto**	8263	8026 (6451; 9518)	8263 (8081, 8449)	8823 (7206, 10338)

Supplementary Table S1.b Excess mortality estimates in 2021 in the different models by region.

	**ISTAT-ISS REPORT 2021**	**Model 1 - 2021**	**Model 2 -2021**	**Model 3 - 2021**
**Abruzzo**	1304	1636 (1047; 2208)	1299 (1256, 1345)	2020 (1731, 2314)
**Basilicata**	515	465 (132; 788)	513 (506, 527)	739 (594, 883)
**Calabria**	2594	2640 (1852; 3411)	2593 (2510, 2680)	2513 (2135, 2895)
**Campania**	6230	7059 (5218; 8877)	6231 (6006, 6464)	7658 (6818, 8486)
**Emilia-Romagna**	4706	4893 (3355; 6406)	4708 (4581, 4838)	6119 (5292, 6925)
**Friuli-Venezia Giulia**	2156	2371 (1853; 2878)	2160 (2059, 2262)	2464 (2173, 2754)
**Lazio**	4851	5486 (3487; 7450)	4858 (4691, 5024)	6358 (5444, 7247)
**Liguria**	591	1022 (249; 1782)	591 (566, 614)	1991 (1591, 2375)
**Lombardia**	8688	7482 (3947; 10954)	8679 (8255, 9137)	10238 (8190, 12110)
**Marche**	2047	2340 (1558; 3098)	2043 (1962, 2135)	2502 (2165, 2841)
**Molise**	568	651 (420; 873)	571 (551, 598)	593 (496, 689)
**Piemonte**	2935	3048 (1058; 5012)	2936 (2752, 3140)	5667 (4672, 6586)
**P.A. Bolzano**	606	600 (339; 850)	603 (577, 630)	569 (460, 679)
**P.A. Trento**	402	392 (117; 657)	402 (377, 427)	618 (500, 741)
**Puglia**	7355	7399 (5834; 8939)	7354 (7026, 7696)	6307 (5646, 6993)
**Sardegna**	1949	1633 (914; 2334)	1948 (1901, 1995)	1679 (1360, 1991)
**Sicilia**	6874	6916 (5145; 8666)	6875 (6588, 7180)	6506 (5683, 7284)
**Toscana**	3443	3736 (2234; 5203)	3441 (3332, 3552)	4978 (4272, 5677)
**Umbria**	1036	1199 (720; 1663)	1038 (1001, 1075)	1427 (1215, 1638)
**Valle d'Aosta**	52	106 (-28; 229)	55 (49, 53)	162 (122, 201)
**Veneto**	4515	4631 (3098; 6151)	4522 (4414, 4626)	5799 (4996, 6556)

Supplementary Table S1.c Relative excess mortality estimates in 2020 in the different models by region.

	ISTAT-ISS REPORT 2020	Model 1 - 2020	Model 2 -2020	Model 3 - 2020
**Abruzzo**	6.14	8.21	6.13	12.43
**Basilicata**	5.33	4.22	5.33	8.8
**Calabria**	3.97	3.83	3.97	6.7
**Campania**	7.04	8.37	7.07	9.15
**Emilia-Romagna**	17.21	17.2	17.21	21.98
**Friuli-Venezia Giulia**	12.47	13.63	12.51	17.17
**Lazio**	5.49	6.25	5.5	8.86
**Liguria**	16.82	18.72	16.85	22.82
**Lombardia**	36.59	34.67	36.44	40.8
**Marche**	12.65	14.17	12.64	17.64
**Molise**	5.91	7.89	5.96	11.27
**Piemonte**	22.89	22.89	23.03	26.98
**P.A. Bolzano**	22.73	22.03	22.73	22.71
**P.A. Trento**	29.91	28.92	29.92	34.24
**Puglia**	12.09	11.89	12.08	12.2
**Sardegna**	12.82	10.44	12.8	10.88
**Sicilia**	5.81	5.42	5.8	7.92
**Toscana**	8.63	9.05	8.63	10.93
**Umbria**	5.56	6.69	5.6	9.46
**Valle d'Aosta**	24.83	28.88	24.88	25.19
**Veneto**	16.67	16.28	16.67	18

Supplementary Table S1.d Relative excess mortality estimates in 2021 the different models by region.

	ISTAT-ISS REPORT 2021	Model 1 - 2021	Model 2 -2021	Model 3 - 2021
**Abruzzo**	8.49	10.93	8.46	13.8
**Basilicata**	7.93	7.13	7.9	11.79
**Calabria**	12.64	12.93	12.64	12.2
**Campania**	11.22	12.95	11.22	14.16
**Emilia-Romagna**	9.24	9.68	9.25	12.36
**Friuli-Venezia Giulia**	14.59	16.34	14.62	17.03
**Lazio**	8.23	9.44	8.24	11.07
**Liguria**	2.67	4.73	2.67	9.61
**Lombardia**	8.71	7.44	8.7	10.43
**Marche**	11.46	13.36	11.44	14.37
**Molise**	14.59	17.13	14.66	15.32
**Piemonte**	5.46	5.7	5.46	11.11
**P.A. Bolzano**	13.63	13.52	13.56	12.69
**P.A. Trento**	7.87	7.69	7.88	12.65
**Puglia**	18.46	18.66	18.46	15.43
**Sardegna**	11.58	9.55	11.57	9.82
**Sicilia**	12.82	12.94	12.82	12.05
**Toscana**	7.77	8.51	7.77	11.64
**Umbria**	9.82	11.58	9.84	14.05
**Valle d'Aosta**	3.5	7.45	3.72	11.82
**Veneto**	9.11	9.39	9.12	12.01
