# Supplementary material for: Assessing COVID-19-Related Excess Mortality Using Multiple Approaches—Italy, 2020–2021
Source: Int J Environ Res Public Health. 2022 Dec 17;19(24):16998. doi: 10.3390/ijerph192416998 (PMC9779266; doi:10.3390/ijerph192416998)
Supplement: Supplementary file 1 [file ijerph-19-16998-s001.zip › Supplementary materials - table S2.pdf]

**Models**	**Estimate 2020**	**Estimate 2021**
ISTAT-ISS [1]	100.526	63.415
Dorrucci [2]	100.530 (96.805, 104.417)	63.421 (61186, 65750)
Maruotti [3]	99.968 (81.474; 117.585)	65.575 (47496; 83478)
Scortichini [4]	116.431 (93146, 136.888)	76.781 (65696, 87079)
WHO [5]	100.431 (90.655, 110.827)	60.370 (55506, 65314)
Konstantinoudis [6]	65.344 (29.416, 97.894)	
Wang [7]	259.000 (242.000, 276.000)	
Levitt (Age-Adjusted) [8]	115.690	
Levitt (non Age-Adjusted) [8]	166.373	
The economist [9]	109.969	75.931

References

[1] ISTAT-ISS, «SETTIMO RAPPORTO: Impatto dell’epidemia Covid-19 sulla mortalità totale della popolazione residente,» 2021. [Online].

[2] M. Dorrucci, G. Minelli, S. Boros, V. Manno, S. Prati, M. Battaglini, G. Corsetti, X. Andrianou, F. Riccardo, M. Fabiani, M. F. Vescio, M. Spuri, A. Mateo-Urdiales, M. Del Manso, G. Onder, P. Pezzotti e A. Bella, «Excess mortality in Italy during the COVID-19 pandemic: assessing the differences between the first and the second wave, year 2020,» *Frontiers in public health,* p. 927, 2021.

[3] A. Maruotti, G. Jona-Lasinio, F. Divino, G. Lovison, M. Ciccozzi e A. Farcomeni, «Estimating COVID-19-induced excess mortality in Lombardy, Italy.,» *Aging clinical and experimental research,* vol. 34.2, pp. 475-479, 2022.

[4] M. Scortichini, R. S. dos Santos, F. de' Donato, M. de Sario, P. Michelozzi, M. Davoli, P. Masselot, F. Sera e A. Gasparrini, «Excess mortality during the COVID-19 outbreak in Italy: a two-stage interrupted time-series analysis.,» *International journal of epidemiology,* vol. 49.6, pp. 1909-1917, 2020.

[5] WHO, «Weekly epidemiological update on COVID-19 4 May 2022,» 2022. [Online]. Available: https://www.who.int/emergencies/diseases/novel-coronavirus-2019/situation-reports.

[6] G. Konstantinoudis, M. Cameletti, V. Gómez-Rubio, I. L. Gómez, M. Pirani, G. Baio, A. Larrauri, J. Riou, M. Egger, P. Vineis e M. Blangiardo, «Regional excess mortality during the 2020,» *Nature communications,* 2022.

[7] H. Wang, «Estimating excess mortality due to the COVID-19 pandemic: a systematic analysis of COVID-19-related mortality, 2020–21.,» *The Lancet,* p. 2022, 2022.

[8] M. Levitt, F. Zonta e J. P. Ioannidis, «Comparison of pandemic excess mortality in 2020-2021 across different empirical calculations,» *medRxiv,* 2022.

[9] T. Economist, «Tracking covid-19 excess deaths across countries,» [Online]. Available: https://www.economist.com/graphic-detail/coronavirus-excess-deaths-tracker. [Consultato il giorno October 2022].
