# Supplementary figures and images for: Assessing COVID-19-Related Excess Mortality Using Multiple Approaches—Italy, 2020–2021
Source: Int J Environ Res Public Health. 2022 Dec 17;19(24):16998. doi: 10.3390/ijerph192416998 (PMC9779266; doi:10.3390/ijerph192416998)

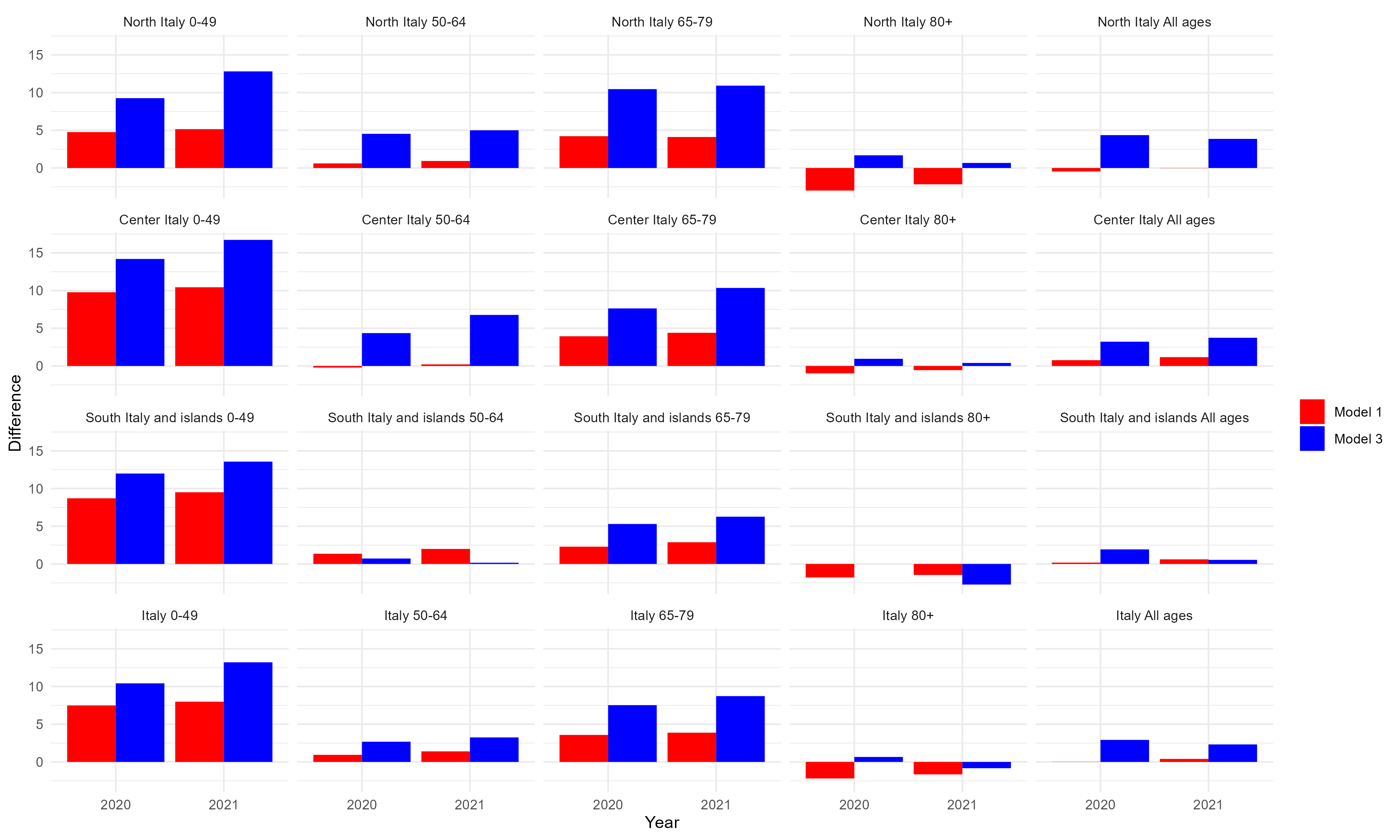

Supplement: Supplementary file 1 [file ijerph-19-16998-s001.zip › Figure S1 - Supplementary materials.png]

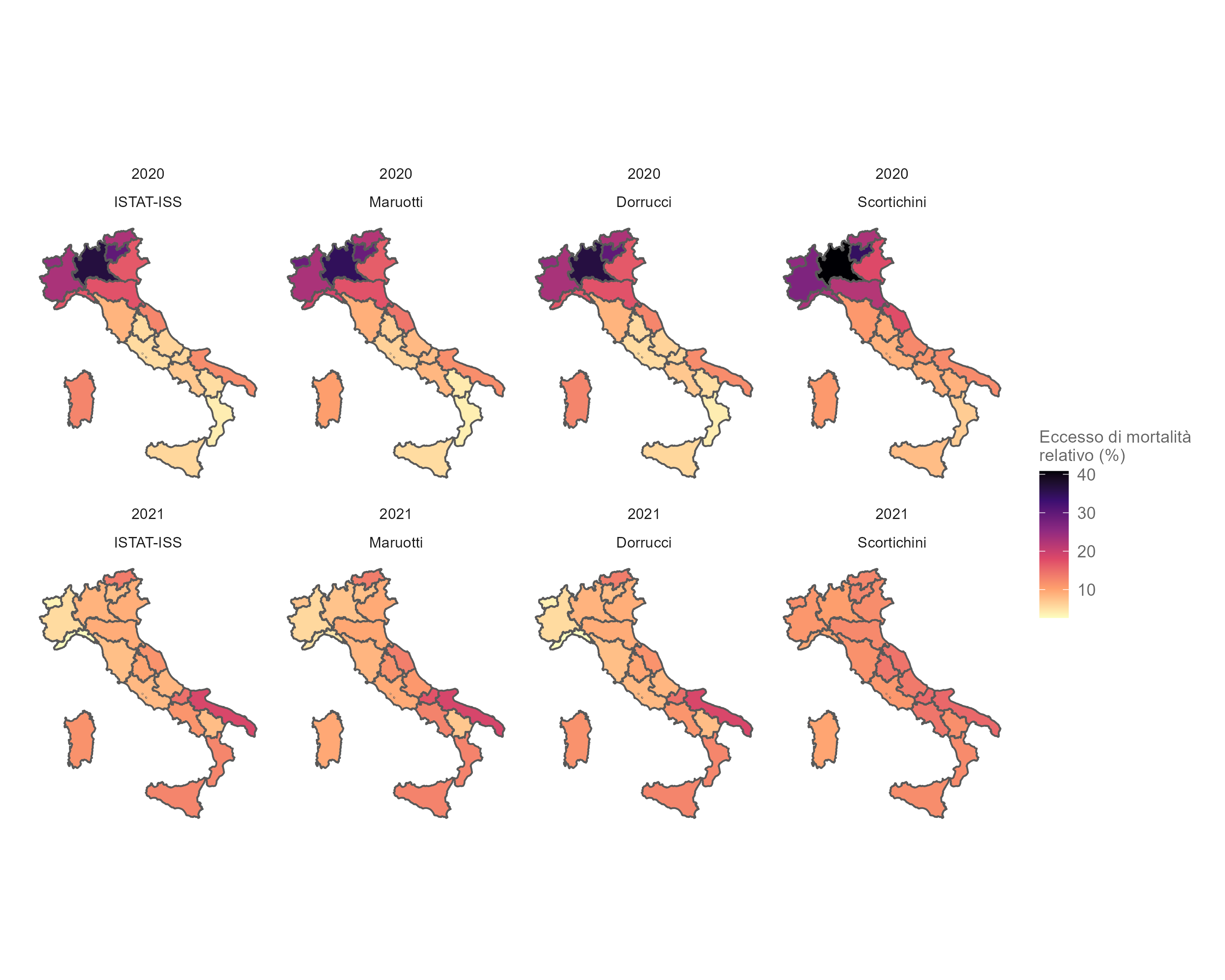

Supplement: Supplementary file 1 [file ijerph-19-16998-s001.zip › Figure S2 - Supplementary materials.png]
